# Agreement of antenatal care indicators from self-reported questionnaire and the antenatal care card of women in the 2015 Pelotas birth cohort, Rio Grande do Sul, Brazil

**DOI:** 10.1186/s12884-019-2573-3

**Published:** 2019-11-08

**Authors:** Lina Sofia Morón-Duarte, Andrea Ramirez Varela, Diego G. Bassani, Andrea Dâmaso Bertoldi, Marlos R. Domingues, Fernando C. Wehrmeister, Mariangela Freitas Silveira

**Affiliations:** 1 Post-Graduate Program in Epidemiology, Federal University of Pelotas, Rua Marechal Deodoro 1160 - Centro, Pelotas, Rio Grande do Sul 96020-220 Brazil; 2 Center for Global Child Health, The Hospital for Sick Children and Department of Pediatrics, University of Toronto, Toronto, Canada

**Keywords:** Agreement, Antenatal care, Indicators, Antenatal card, Self-reported questionnaire

## Abstract

**Background:**

Studies of healthcare service use during the pregnancy-postpartum cycle often rely on self-reported data. The reliability of self-reported information is often questioned as administrative data or medical records, such as antenatal care cards, are usually preferred. In this study, we measured the agreement of antenatal care indicators from self-reported information and antenatal care cards of pregnant women in the 2015 Pelotas Birth Cohort, Brazil.

**Methods:**

In a sample of 3923 mothers, indicator agreement strengths were estimated from Kappa and prevalence-and-bias-adjusted Kappa (PABAK) coefficients. Maternal characteristics associated with indicator agreements were assessed with heterogeneity chi-squared tests.

**Results:**

The self-reported questionnaire and the antenatal care card showed a moderate to high agreement in 10 of 21 (48%) antenatal care indicators that assessed care service use, clinical examination and diseases during pregnancy. Counseling indicators performed poorly. Self-reported information presented a higher frequency data and a higher sensitivity but slightly lower specificity when compared to the antenatal card. Factors associated with higher agreement between both data sources included lower maternal age, higher level of education, primiparous status, and being a recipient of health care in the public sector.

**Conclusions:**

Self-reported questionnaire and antenatal care cards provided substantially different information on indicator performance. Reliance on only one source of data to assess antenatal care quality may be questionable for some indicators. From a public health perspective, it is recommended that antenatal care programs use multiple data sources to estimate quality and effectiveness of health promotion and disease prevention in pregnant women and their offspring.

## Background

Antenatal Care (ANC) comprises a number of activities and procedures aimed at preserving the health of pregnant women while ensuring delivery of preventive measures, early detection of complications and the adequate management of pre-existing maternal disease states [1]. The reduction of maternal mortality remains a priority under “Goal 3: Ensure healthy lives and promote well-being for all at all ages” in the new Sustainable Development Goals (SDGs) agenda through 2030 [2]. In this context, information on maternal and child health is fundamental to ensure antenatal care quality and reduction of maternal and perinatal mortality. In Brazil, a pregnancy card records health information and details of antenatal and postpartum care visits. The card is a tool included in the Prenatal and Birth Humanization Program, part of the Ministry of Health medical record systems, and its use is mandatory to ensure that healthcare for pregnant women meets the national minimum quality standards. The card includes information on ANC visits, diagnostic findings and patient behaviors and links the different stages of the health care process. The systematic collection of data during each consultation can therefore be used to provide optimal health services at any point of care.

An alternative method for collection of data on health care received during the pregnancy-postpartum cycle is the use of individual questionnaires. The in-person interview or questionnaire is frequently used in epidemiology research because it provides a comprehensive and standardized assessment of the topic of interest. It is a relatively inexpensive tool that enables collection of data from a large population sample.

Medical records are considered the gold standard and are generally the preferred data source over interviews/questionnaires. However, evidence indicates that reliability of any individual source of data is imperfect, possibly inaccurate, and thus the combination of sources may improve reliability and completeness of information [1, 3–6]. The objective of this study was to evaluate for the first time the agreement between self-reported questionnaire information and data recorded in the ANC cards from women in the 2015 city of Pelotas Birth Cohort study, Rio Grande do Sul, Brazil.

## Materials and methods

### Study settings

The data is representative of births in the municipality of Pelotas in 2015. However, this birth cohort is not representative of the total number of births in Brazil for that period. In 2015, 5598 newborn babies were identified, of whom 4329 were children of mothers who were residents in the urban area of Pelotas (cohort study targeted population) and were invited to participate. The refusal rate was 1.3% in the perinatal period.

### Sampling

This was a cross-sectional study based on the mothers of children eligible for the 2015 Pelotas Birth Cohort who were interviewed during pregnancy and/or at birth. The total number of eligible pregnant mothers was 4329 (in the case of multiple pregnancies only one record was kept for each mother). Fifty-nine records corresponded to multiple births, totaling 4270 mothers in the birth cohort. Of these mothers 4172 had attended ANC but 249 did not have an antenatal card available at the time of interview, resulting in a final sample of 3923 mothers included in this study (Additional file 1: Figure S1).

The questionnaires used during prenatal and perinatal visits of the 2015 Pelotas Birth Cohort are available at http://epidemio-ufpel.org.br/site/content/coorte_2015/questionarios.php. More detailed information on the cohort methods and follow-up visits is provided elsewhere [7].

### Variables

The information was collected through interviews during pregnancy and/or the perinatal period. During antenatal care, two antenatal visits were performed, one before gestational week 16 and the second between gestational weeks 16 and 24. The third follow-up was at delivery. One questionnaire was used at weeks 16, 24 and at delivery to collect sociodemographic characteristics including maternal age, education, marital status, skin color, family income, pre-pregnancy health, and if the mother was seen by the same health professional during ANC and type of ANC provider. The perinatal questionnaire asked for reproductive health history variables – delivery characteristics, number of pregnancies, and if the mother was seen by the same health professional during ANC and type of ANC provider. ANC data were also collected at delivery and documented service utilization, details of clinical examination, occurrence of disease during pregnancy, prophylactic interventions (vaccination and supplements), and educational interventions (counseling). Additional file 2: Table S1 shows the questionnaire contents and data collection tools.

At the end of the interview the ANC card was photographed and information on ANC routines extracted and double entered into a database (Epi Info 6.04, Centers for Disease Control and Prevention, Atlanta, USA) by two trained research assistants. A consistency analysis with a frequency verification was performed using Stata 12® (Statacorp, College Station, TX, USA).

If there was no record of a given procedure on the card, it was considered as not performed. The agreement analysis was carried out only when the card was available with at least one record present. The questionnaire items and antenatal card variables used in this study are described in Additional file 3: Table S2.

### Statistical analysis

The agreement between the card and the questionnaire for each variable of interest was estimated using Kappa coefficient (κ) with corresponding 95% confidence intervals (95% CI). Kappa and Prevalence-Adjusted-and-Bias-Adjusted Kappa (PABAK) [8] values were interpreted using Landis and Koch categorization [9] into almost perfect (>0.80), substantial (0.61–0.80), moderate (0.41–0.60), fair (0.21–0.40), slight (0.00–0.20) and poor (<0.00). Sensitivity and specificity are reported with 95% CI and were calculated assuming the ANC card to be the gold standard or assuming the questionnaire to be the gold standard.

The association of maternal characteristics with an agreement between the card and the questionnaire was evaluated by building a score ranging from 1 to 10 using those ANC indicators with adjusted Kappa coefficients in the categories of almost perfect to moderate (ten indicators as shown in Table 3). A value of 1 was assigned to each indicator agreement (1 = yes/yes or no/no). The score was dichotomized into low (≤7 points as zero) and high agreement (≥ 8 points as one) to conduct bivariate analysis using the score as the dependent variable. Heterogeneity chi-squared tests were used to measure the difference between low and high agreement categories. The analyses were performed in Stata 15® (Statacorp, College Station, TX, USA).

## Results

### Sample characteristics

Within the cohort of 3923 women 82.6% were between 20 and 39 years of age, 14.4% were under 20 years of age, 71.9% were white, 33.7% had up to 8 years of schooling and 86.4% were living with a partner at the time of interview. With regard to socioeconomic data, 59.7% were between the first and third quintiles of family income. In relation to obstetrical data, 50.4% of the women were primiparous, 64.8% had undergone c-section delivery, 52.8% were seen by the same health professional during ANC, and 45.0% received ANC in the public health sector. When comparing our sample with mothers who did not attend or receive ANC and lacked an antenatal card, a statistically significant difference for maternal education, marital status, family income, and parity was found (Table 1). Table 2 shows the distribution of data from ANC cards and from the self-reported questionnaire.

**Table 1:** Maternal characteristics in the 2015 Pelotas Birth Cohort, Brazil

Characteristics	Mothers with ANC and card (*n* = 3923) n (%)	Mothers without ANC or card (*n* = 346) n (%)	Chi-squared test *p* value
Age (years)			
≤ 19	565 (90.0)	63 (10.0)	0.144
20–29	1.878 (92.7)	147 (7.3)	
30–39	1.365 (91.6)	125 (8.4)	
≥ 40	115 (91.3)	11 (8.7)	
Maternal education (complete years of schooling)			
0–4	337 (85.5)	57 (14.5)	<0.001
5–8	985 (89.7)	113 (10.3)	
9–11	1.388 (94.9)	75 (5.1)	
12 +	1.213 (92.3)	101 (7.7)	
Marital status			
Without partner	535 (86.4)	84 (13.6)	<0.001
With partner	3.388 (92.8)	262 (7.2)	
Skin color			
White	2.824 (92.5)	230 (7.5)	0.086
Black	586 (90.2)	64 (9.8)	
Other	513 (90.8)	52 (9.2)	
Family income (quintiles)			
Lowest/first	759 (88.5)	99 (11.5)	
Second	784 (91.8)	70 (8.2)	**<**0.001
Third	802 (93.6)	55 (6.4)	
Fourth	803 (93.7)	54 (6.3)	
Highest/fifth	774 (91.9)	68 (8.1)	
Type of delivery			
Normal	1.378 (91.2)	133 (8.8)	0.217
C-section	2.545 (92.3)	213 (7.7)	
Parity			
Primiparous	1.977 (93.5)	137 (6.5)	<0.001
≥ 2 children	1.945 (90.3)	209 (9.7)	
ANC consultation by same professional			
Yes	2.074 (93.4)	147 (6.6)	0.059
No	1.849 (94.8)	102 (5.2)	
Type of health care provider			
Public	1.372 (98.6)	20 (1.4)	0.175
Private	1.203 (97.6)	30 (2.4)	
Others	472 (97.9)	10 (2.1)	

For some variables, the number of subjects does not add up to the total of the subjects included due to missing information

**Table 2:** Distribution of ANC indicator data by information source

	Source			
	ANC card		Self-reported	
Indicator	Yes n (%)	No n (%)	Yes n (%)	No n (%)
Report of the number of antenatal care visits (≥6)	2703 (71.1)	1101 (28.9)	3373 (86.3)	536 (13.7)
Report of the last date of menstrual period	2852 (72.7)	1071 (27.3)	3887 (99.1)	35 (0.9)
Clinical exams				
Weight measurement	3781 (96.4)	142 (3.6)	3914 (99.8)	8 (0.2)
Symphysis-fundal height measurement	3666 (93.5)	257 (6.5)	3903 (99.5)	19 (0.5)
Blood pressure measurement	3782 (96.4)	141 (3.6)	3921 (99.9)	2 (0.1)
Gynecological exam	1391 (35.5)	2534 (64.5)	3355 (85.5)	567 (14.5)
Cervical cancer screening test	737 (18.8)	3186 (81.2)	2526 (64.5)	1388 (35.5)
Dental exam	186 (4.7)	3737 (95.3)	1412 (36.0)	2510 (64.0)
Breast exam	657 (16.8)	3266 (83.3)	1821 (46.5)	2096 (53.5)
Venereal disease research laboratory (VDRL) test	3579 (91.2)	344 (8.8)	3916 (99.8)	7 (0.2)
Diseases during pregnancy				
Hypertension	43 (1.1)	3880 (98.9)	1005 (25.6)	2917 (74.4)
Anaemia	136 (3.8)	3288 (96.3)	1662 (42.4)	2259 (57.6)
Diabetes	24 (0.6)	3899 (99.4)	348 (8.9)	3575 (91.1)
Urinary tract infection	1256 (32.0)	2667 (68.0)	1782 (45.5)	2135 (54.5)
Sexually transmitted diseases	111 (2.8)	3812 (97.2)	38 (1.0)	3881 (99.0)
Vaccination-Supplements				
Tetanus toxoid vaccination	2102 (53.6)	1821 (46.4)	2473 (64.0)	1393 (36.0)
Hepatitis B vaccination	1312 (33.4)	2611 (66.5)	1973 (51.4)	1869 (48.7)
Iron supplements prescription	991 (25.3)	2932 (74.7)	3100 (79.0)	823 (21.0)
Counselling				
Risks of alcohol use during pregnancy	38 (1.0)	3885 (99.0)	2919 (74.5)	1001 (25.5)
Risks of smoking during pregnancy	46 (1.2)	3877 (98.8)	2947 (75.2)	973 (24.8)
Physical exercises such as walking	3 (0.1)	3920 (99.9)	2430 (62.0)	1491 (38.0)

### Agreement between ANC card indicators and questionnaire information

From the 21 indicators in the ANC card we observed that agreement strength with self-reported questionnaire information was poor in 1 indicator (K < 0.00), slight for 16 (K between 0.00–0.20), fair for 2 (K between 0.21–0.40), and moderate for 2 (K between 0.41–0.60) (Table 3). The agreement strength category obtained with the PABAK was higher than that obtained with the Kappa coefficient in 9 (43%) indicators – antenatal care number of visits ≥6, reported date of last menstrual period, weight, symphysis-fundal height, blood pressure, syphilis test, hypertension during pregnancy, diabetes during pregnancy, and sexually transmitted diseases (Table 3). Agreement strength categories for urinary tract infection during pregnancy, breast exam, hepatitis B vaccination, and counseling on physical activities such as walking were moderate, slight, fair, and poor respectively, and were equally categorized by PABAK and Kappa values. Agreement strength categories were lower with PABAK relative to those obtained with Kappa for gynecological exam, cervical cancer screening test, counseling on risks of alcohol use during pregnancy and counseling on risks of smoking during pregnancy (Table 3).

**Table 3:** Concordance measures of ANC indicators from the ANC card and self-reported questionnaire information

	Concordance ANC card/Self-reported		Discordance ANC card/Self-reported		% Agreement (95% CI)	Kappa (95% CI)	*P* value	Agreement strength category^a^	PABAK (95% CI)	Agreement strength category^b^	*P* value
	No/No	Yes/Yes	Yes/No	No/Yes							
Indicator	% (n)	% (n)	% (n)	% (n)							
Report of the number of antenatal care visits^d^ (*n* = 3791)	12.6 (479)	70.1 (2657)	1.0 (36)	6.3 (619)	82.72 (81.43–84.02)	0.50 (0.47–0.53)	<0.001	moderate	0.65 (0.63–0.68)	substantial	<0.001
Report of the last date of menstrual period (*n* = 3922)	0.4 (16)	72.2 (2832)	0.5 (19)	26.9 (1055)	72.62 (71.22–74.01)	0.01 (0.00–0.02)	0.043	slight	0.45 (0.42–0.48)	moderate	<0.001
Clinical exams											
Weight measurement (*n* = 3922)	0.0 (1)	96.2 (3773)	0.2 (7)	3.6 (141)	96.23 (95.63–96.82)	0.01(−0.02–0.03)	0.472	slight	0.92 (0.91–0.94)	almost perfect	<0.001
Symphysis-fundal height measurement (*n* = 3922)	0.2 (8)	93.2 (3654)	0.3 (11)	6.4 (249)	93.37 (92.59–94.15)	0.05 (0.01–0.08)	0.009	slight	0.87 (0.85–0.88)	almost perfect	<0.001
Blood pressure measurement (*n* = 3923)	0.0 (0)	96.4 (3780)	0.1 (2)	3.6 (141)	96.35 (95.77–96.94)	^c^-0.00(−0.00–0.00)	0.152	poor	0.96 (0.96–0.97)	almost perfect	<0.001
Gynecological exam (*n* = 3922)	10.5 (413)	31.5 (1237)	3.9 (154)	54.0 (2118)	42.07 (40.52–43.62)	0.04 (0.02–0.05)	<0.001	slight	^c^-0.16(−0.19- -0-13)	poor	<0.001
Cervical cancer screening test (*n* = 3914)	31.7 (1241)	15.0 (587)	3.8 (147)	49.5 (1939)	46.70 (45.14–48.27)	0.09 (0.07–0.11)	<0.001	slight	^c^-0.07(−0.09- -0.03)	poor	<0.001
Dental exam (*n* = 3922)	61.0 (2391)	1.7 (67)	3.0 (119)	34.3 (1345)	62.67 (61.16–64.19)	0.00(−0.02–0.02)	0.996	slight	0.25 (0.22–0.28)	fair	<0.001
Breast exam (*n* = 3917)	45.9 (1796)	9.1 (357)	7.7 (300)	37.4 (1464)	54.97 (53.41–56.53)	0.05 (0.03–0.08)	<0.001	slight	0.09 (0.07–0.13)	slight	<0.001
Venereal disease research lab (VDRL) test (*n* = 3923)	0.0 (1)	91.1 (3573)	0.2 (6)	8.7 (343)	91.10 (90.21–91.99)	0.00(−0.01–0.01)	0.68	slight	0.82 (0.80–0.84)	almost perfect	<0.001
Diseases during pregnancy											
Hypertension (*n* = 3922)	74.0 (2903)	0.7 (29)	0.4 (15)	24.9 (976)	74.76 (73.40–76.12)	0.03 (0.02–0.05)	<0.001	slight	0.49 (0.47–0.52)	moderate	<0.001
Anaemia (*n* = 3921)	57.2 (2241)	3.0 (118)	0.5 (18)	39.4 (1544)	60.16 (58.63–61.70)	0.07 (0.06–0.09)	< 0.001	slight	0.20 (0.17–0.23)	fair	<0.001
Diabetes (*n* = 3923)	90.9 (3567)	0.4 (16)	0.2 (8)	8.5 (332)	91.33 (90.45–92.21)	0.07 (0.04–0.11)	<0.001	slight	0.82 (0.81–0.84)	almost perfect	0.001
Urinary tract infection (*n* = 3917)	47.6 (1866)	25.2 (986)	6.9 (269)	20.3 (796)	72.81 (71.41–74.21)	0.41 (0.41–0.47)	<0.001	moderate	0.46 (0.43–0.48)	moderate	<0.001
Sexually transmitted diseases (*n* = 3919)	96.3 (3774)	0.1 (2)	2.7 (107)	0.9 (36)	96.35 (95.76–96.95)	0.02(−0.02–0.07)	0.218	slight	0.93 (0.92–0.94)	almost perfect	<0.001
Vaccination-Supplements											
Tetanus toxoid vaccination (*n* = 3866)	21.5 (831)	39.1 (1513)	14.5 (562)	24.8 (960)	60.63 (59.07–62.19)	0.19 (0.17–0.22)	<0.001	slight	0.21 (0.18–0.24)	fair	<0.001
Hepatitis B vaccination (*n* = 3842)	38.8 (1490)	23.8 (913)	9.9 (379)	27.6 (1060)	62.55 (60.99–64.10)	0.26 (0.23–0.29)	<0.001	fair	0.25 (0.22–0.28)	fair	<0.001
Prescription of iron supplements (*n* = 3923)	17.8 (697)	22.1 (865)	3.2 (126)	57.0 (2235)	39.82 (38.28–41.35)	0.06 (0.05–0.08)	<0.001	slight	^c^-0.20(−0.23- -0.17)	poor	<0.001
Counselling											
Risks of alcohol use during pregnancy (*n* = 3920)	25.4 (994)	0.8 (31)	0.2 (7)	73.7 (2888)	26.15 (24.77–27.52)	0.00(−0.00–0.01)	0.263	slight	^c^-0.48(−0.50- -0.45)	poor	<0.001
Risks of smoking during pregnancy (*n* = 3920)	24.6 (965)	1.0 (38)	0.2 (8)	74.2 (2909)	25.59 (24.22–26.95)	0.00(−0.00–0.01)	0.189	slight	^c^-0.49(−0.52- -0.46)	poor	<0.001
Physical exercises such as walking (*n* = 3921)	38.0 (1489)	0.0 (1)	0.1 (2)	62.0 (2429)	38.00 (36.48–39.52)	^c^-0.00(−0.00- -0.00)	0.368	poor	^c^-0.24 (−0.27- -0.21)	poor	<0.001

^a^According to Landis and Koch categorization as almost perfect (>0.80), substantial (0.61–0.80), moderate (0.41–0.60), fair (0.21–0.40), slight (0.00–0.20) and poor (<0.00)

^b^Strength of agreement of coefficient PABAK according to Landis and Koch

^c^negative estimate

^d^> =6 Visit

### Sensitivity and specificity

#### Self-reported questionnaire with the ANC card as the gold standard

The sensitivity of the self-reported questionnaire was high (>90%) for 28.6% of the indicators evaluated, all of them in the components of service use and clinical examination. Good sensitivity (≥80 and < 90%) was present in 23.8% and low sensitivity (<80%) in 47.6% of the indicators. Specificity was low (<80%) for all indicators except for diabetes and sexually transmitted diseases that had >90% specificity (Table 4.).

**Table 4:** Validity of ANC indicators from self-reported questionnaire information or the antenatal card

Indicators	Self-reported with ANC card as the gold standard				ANC card with self-reported as the gold standard			
	Sensitivity (%)	95% CI	Specificity (%)	95% CI	Sensitivity (%)	95% CI	Specificity (%)	95% CI
Report of the number of antenatal care visits (≥6)	98.66	98.21–99.12	43.62	40.65–46.60	81.11	79.75–82.46	93.01	90.71–95.31
Report of the last date of menstrual period	99.33	99.02–99.65	1.49	0.72–2.27	72.86	71.45–74.27	45.71	27.78–63.65
Clinical exams								
Weight measurement	99.81	99.66–99.97	0.70	0.02–3.86	96.40	95.80–96.99	12.5	0.31–41.67
Symphysis-fundal height measurement	99.71	99.52–99.89	3.11	0.80–5.43	93.62	92.84–94.40	42.11	17.27–66.94
Blood pressure measurement	99.90	99.80–100.00	0.00		96.40	95.80–97.01	0.00	
Gynecological exam	88.93	87.24–90.61	16.32	14.86–17.78	36.87	35.22–38.52	72.84	69.02–76.59
Cervical cancer screening test	79.97	77.01–82.94	39.03	37.31–40.74	23.24	21.57–24.91	89.41	87.75–91.06
Dental exam	36.02	28.85–43.19	64.00	62.45–65.55	4.75	3.60–5.89	95.26	94.41–96.11
Breast exam	54.34	50.45–58.22	55.09	53.37–56.81	19.60	17.75–21.46	85.69	84.16–87.21
Venereal disease research lab (VDRL) test	99.83	99.68–99.98	0.29	0.01–1.61	91.24	90.34–92.14	14.29	0.36–47.35
Diseases during pregnancy								
Hypertension	67.44	52.27–82.61	74.84	73.46–76.22	2.89	1.80–3.97	99.52	99.25–99.79
Anaemia	86.76	80.70–92.83	59.21	57.63–60.79	7.10	5.84–8.36	99.20	98.81–99.59
Diabetes	66.67	45.72–87.61	91.48	90.60–92.37	4.60	2.25–6.94	99.78	99.61–99.95
Urinary tract infection	78.57	76.26–80.88	70.10	68.34–71.86	55.33	52.99–57.67	87.40	85.97–88.83
Sexually transmitted diseases	1.83	0.00–4.81	99.06	98.73–99.38	5.26	0.64–13.68	97.24	96.71–97.77
Vaccination- Supplements								
Tetanus toxoid vaccination	78.57	76.26–80.88	70.10	68.34–71.86	61.18	59.24–63.12	59.66	57.04–62.27
Hepatitis B vaccination	70.67	68.14–73.19	58.43	56.50–60.36	46.27	44.05–48.50	79.72	77.87–81.57
Prescription of iron supplements	87.29	85.16–89.41	23.77	22.21–25.33	27.90	26.31–29.50	84.69	82.17–87.21
Counselling								
Risks of alcohol use during pregnancy	81.58	67.94–95.22	25.61	24.22–26.99	1.06	0.67–1.45	99.30	98.73–98.87
Risks of smoking during pregnancy	82.61	70.57–94.65	24.91	23.53–26.28	1.29	0.87–1.71	99.18	98.56–99.80
Physical exercises such as walking	33.33	0.84–90.60	38.00	36.47–39.54	0.04	0.00–0.14	99.87	99.65–100.00

#### Antenatal card with the self-reported questionnaire as the gold standard

The sensitivity of antenatal card was high (>90%) for 19.0% of the indicators evaluated, all of them in the clinical examination component. The number of visits showed good sensitivity (81.1%) and the rest presented low sensitivity. Specificity was high for 42.8% of the indicators, mainly those related to diseases during pregnancy and counselling. Good specificity (≥80 and < 90%) was present in 19.0% of the indicators (Table 4).

#### Maternal factors associated with an agreement between data sources

Table 5 presents the maternal characteristics associated with the concordance between the ANC card and self-report questionnaire. There was a significantly higher agreement among pregnant women with younger age, more years of maternal schooling, primiparous status and those using a public sector health care provider.

**Table 5:** Maternal characteristics associated with agreement between the ANC card and self-reported questionnaire

Characteristics	Low n (%)	High n (%)	Chi-squared test *p* value
Age (years)			
≤ 19	85 (15.0)	480 (85.0)	<0.001
20–29	272 (14.5)	1606 (85.5)	
30–39	252 (18.5)	1113 (81.5)	
≥ 40	38 (33.0)	77 (67.0)	
Maternal education (complete years of schooling)			
0–4	76 (22.6)	261 (77.5)	0.008
5–8	169 (17.2)	816 (82.8)	
9–11	207 (14.9)	1181 (85.1)	
12 +	195 (16.1)	1018 (83.9)	
Marital status			
Without partner	99 (18.5)	436 (81.5)	0.177
With partner	548 (16.2)	2840 (83.8)	
Skin color			
White	459 (16.3)	2365 (83.8)	0.523
Black	106 (18.1)	480 (81.9)	
Other	82 (16.0)	431 (84.0)	
Family income			
Lowest/first	144 (19.0)	615 (81.0)	0,062
Second	118 (15.1)	666 (85.0)	
Third	125 (15.6)	677 (84.4)	
Fourth	147 (18.3)	656 (81.7)	
Highest/fifth	113 (14.6)	661 (85.4)	
Type of delivery			
Normal	206 (15.0)	1172 (85.1)	0.055
C-section	441 (17.3)	2104 (82.7)	
Parity			
Primiparous	298 (15.1)	1679 (84.9)	0.017
≥ 2 children	348 (17.9)	1597 (82.1)	
The same professional performed the antenatal consultations			
Yes	341 (16.4)	1733 (83.5)	0.928
No	306 (16.6)	1543 (83.5)	
Type of health care provider			
Public sector	192 (14.0)	1180 (86.0)	0.005
Private sector	194 (16.1)	1009 (83.9)	
Others	96 (20.3)	376 (79.7)	

## Discussion

### Main findings

To our knowledge, this is one of the few studies evaluating the agreement between self-reported questionnaire from pregnant women and the antenatal card that recorded procedures and interventions during ANC [10, 11]. The assessment of agreement between sources of information on ANC assistance is an issue that has aroused the interest of the global scientific community. Our key findings indicate firstly, that assessment of ANC from self-reported questionnaire has moderate to high agreement to ANC card indicators of service utilization, clinical examination and diseases during pregnancy whereas indicators of counseling performed poorly. Secondly, self-reported information presented higher data frequency and higher sensitivity but slightly lower specificity when compared to the ANC card. Thirdly, factors associated with higher agreement between both data sources included young maternal age, more maternal schooling years, being a primiparous mother, and health care received in the public sector.

#### Moderate to high agreement on indicators of service utilization, clinical examination and diseases during pregnancy. Poor agreement on indicators of counseling

The moderate to high agreement in these indicators suggests that assessment of ANC through self-reported questionnaire or the antenatal card could be equivalent. However, caution is advised when interpreting these results, especially for indicators related to diseases during pregnancy. As has been shown in other studies [12], the performance of antenatal clinical exams such as measurements of weight, symphysis-fundal height, and blood pressure reached almost perfect agreement [10, 12]. These findings may be related to the ability of the patient to identify the reason for the ANC procedure/action recorded by the health professional during the ANC visit. For example, self-reporting weight may be more accurate than self-reporting a cervical cancer screening test. Patients are more likely to understand that weight is being measured when asked to step on a scale but less likely to discern whether a cervical cancer screening test is the reason for a gynecological examination [13–15]. Indeed we found a very low agreement on reporting of gynecological exam, cervical cancer screening test and breast exam (Table 3). Given that the frequency of these procedures was lower in the ANC cards relative to self-reports, the low agreements may also stem from poor record-keeping by health professionals or that these tests, that are mandatory at the national level, were not carried out, as reported in other studies [10–14, 16–23]. This would raise serious concerns regarding the quality of healthcare that women and their offspring receive.

Efforts to improve the quality of care for pregnant women in Brazil include a National Oral Health Policy [24] which mandates a dental consultation when initiating ANC. Our study found a fair agreement. However, the results should be analyzed with caution as 70.9% (1002/1412) of the pregnant women who reported having consulted with a dentist during pregnancy also participated in the oral health sub-study of this cohort [7], therefore in the general population agreement could be lower than reported.

For gestational diabetes, agreement was classified as almost perfect, a finding confirmed by other studies [25, 26] where agreement was often higher than those reported for other chronic diseases [27, 28]. Studies evaluating self-reported medical information have shown that patients can provide reasonably good reports on their illnesses [29, 30]. Recent publications on urinary tract infection have shown moderate agreement between self-reporting and medical records with a low sensitivity [31, 32], similar to the findings obtained by our study.

Some conditions such as diabetes, hypertension, anemia, and urinary tract infection may not have been recorded on the ANC cards, but women still refer to having experienced them. Similar findings have been reported in other studies [10, 33, 34]. One possible reason for over reporting of gestational diabetes is that the diagnosis requires a positive result on two different glucose tests. Thus, women with a positive first test but negative on the second may have reported gestational diabetes. Sexually transmitted diseases may have been under-reported possibly due to stigmatization.

Counselling indicators were more frequently self-reported than reported in the antenatal card. Reports of counselling pertaining to alcohol use and smoking during pregnancy had the lowest Kappa values and the highest difference between sources, perhaps due to a low probability of reporting of these habits to healthcare providers during antenatal care. Previous studies have shown that counseling and referrals (e.g., cervical cancer screening tests) are underreported in medical records compared to self-reported records, indicating that health professionals do not consistently register these interventions [35, 36].

#### . Self-reported information presented higher data frequency and higher sensitivity but slightly lower specificity when compared to the ANC card

The use of questionnaires in epidemiologic studies is considered a valid tool with many advantages for research. However, the quality of the information obtained by self-reporting is dependent on the type of disease [37, 38], the characteristics of the participants [30, 39], the design of the questionnaire, and the method used to administer it [40, 41]. In addition, information obtained by self-reporting or by review of medical records may not be consistent. Several studies have shown that the agreement between the two sources of information is dependent on the type of variable collected [42–44].

When self-report was compared to the card as gold standard, we found that specificity and sensitivity were > 80% for 9.5% (2/21) and 52% (11/51) of ANC indicators respectively. The high sensitivity in this comparison may be related to the use of self-administered questionnaires as sources of information on exposures and outcomes, which may be more complete and with fewer omissions in responses by participants.

When we compared the antenatal card with self-report as the gold standard, we found that the specificity and sensitivity were > 80% for 60% (13/21) and 25% (5/21) of ANC indicators respectively. This high specificity suggests that data recorded on the cards corresponded to the most relevant aspects identified during medical consultation. The low sensitivity suggests that registration of other ANC procedures/activities was incomplete. One reason may be that data recorded on antenatal cards do not accurately reflect all events occuring during a medical visit [45, 46]. Furthermore the content of antenatal cards is not standardized for data collection in a private versus public setting or designed for data collection by health professionals with responsibility for specific aspects of ANC.

Despite these limitations, data collected in the ANC card are preferable for making decisions on potentially significant clinical interventions during antenatal and perinatal stages [47].

#### . Maternal characteristics associated with the agreement between the ANC card and the self-reported questionnaire

Sociodemographic characteristics may contribute towards the strength of agreement between data sources and determine ANC quality [48]. Our findings show that higher maternal age, lower educational status, pregnant women with ≥2 children and type of healthcare provider associate with a lower probability of high agreement between the antenatal card and the self-reported questionnaire. We were unable to find other studies on factors associated with ANC indicator agreements for the data sources used in our study. However, there is evidence that the demand for health services and ANC quality are typically related to the sociodemographic characteristics of pregnant women [49].

### Limitations and implications

Due to the intrinsic limitations of the data sources analyzed herewith, judgements about their respective validity should be made with caution.

The quality of medical record documentation may be affected by omissions in reporting, for example tests may have been performed elsewhere and not transcribed onto the antenatal card or incomplete documentation due to time constraints for record-keeping by health professionals. In addition to problems with recording-keeping, accuracy of medical record extraction may be compromised by bias, fatigue or distraction of the systematic reviewer. These findings suggest that a purely quality-based assessment on medical records data could fail to find information on performed procedures and interventions that the pregnant woman might have supplied. This can result in the loss of important information on some ANC indicators.

The quality of an evaluation that depends solely on self-reported information can be influenced by recall bias, by subjectivity or misunderstanding of the questions pertaining to diagnoses, procedures and interventions received during ANC. In addition, the accuracy of a self-report may be affected by culturally influenced factors such as the importance of events, awareness and knowledge of health conditions [50].

## Conclusions

Our study shows that data from questionnaires or the ANC cards provide substantially different information on indicator performance. From a public health perspective this raises questions with regard to reliance on indicators derived from a single data source. It may be more prudent to assess the quality of ANC programs using multiple data sources to determine quality and effectiveness of health promotion and disease prevention programs in pregnant women and their offspring. Researchers should explore alternative methods and data sources to obtain consistent estimates of ANC quality, various different indicators, be aware of factors that may influence the accuracy of data sources and conduct sub-studies to collect such data when not available. The implications of choosing a questionnaire or medical records should be carefully considered when evaluating health services for clinical practice, research, and public health. Deciding which data source to use will also depend on the outcome of interest and if the data is used for clinical decision-making, performance tracking, or public health.

## Supplementary information

**Additional file 1:** **Figure S1.** Flowchart sample.

**Additional file 2:** **Table S1.** Questionnaire contents and data collection tools, Pelotas 2015.

**Additional file 3:** **Table S1.** Questionnaire items and the antenatal care card variables.

## Data Availability

The dataset used in this study is available upon request.

## Abbreviations

ANC: Antenatal care
CI: Confidence interval
PABAK: Prevalence-Adjusted-and-Bias-Adjusted Kappa
SDGs: Sustainable Development Goals
