# Supplementary material for: Agreement of antenatal care indicators from self-reported questionnaire and the antenatal care card of women in the 2015 Pelotas birth cohort, Rio Grande do Sul, Brazil
Source: BMC Pregnancy Childbirth. 2019 Nov 8;19:410. doi: 10.1186/s12884-019-2573-3 (PMC6839160; doi:10.1186/s12884-019-2573-3)
Supplement: Supplementary file 1 — Additional file 1: Figure S1. Flowchart sample. [file 12884_2019_2573_MOESM1_ESM.docx]

Figure 1. Flowchart sample

Eligible births for inclusion in the 2015 Pelotas birth cohort study

n=4329

If there were multiple pregnancies, only one record was kept for each mother. Fifty-nine records corresponded to multiple births n=59

Pelotas birth cohort mothers in 2015

n=4270

Mothers who did not attend or receive antenatal care in 2015

n=98

Pelotas birth cohort mothers that had antenatal care in 2015 n=4172

Mothers who did not have an antenatal card in 2015

n=249

Pelotas birth cohort mothers that had antenatal care and an antenatal card in 2015

n=3923
