# Supplementary material for: Agreement of antenatal care indicators from self-reported questionnaire and the antenatal care card of women in the 2015 Pelotas birth cohort, Rio Grande do Sul, Brazil
Source: BMC Pregnancy Childbirth. 2019 Nov 8;19:410. doi: 10.1186/s12884-019-2573-3 (PMC6839160; doi:10.1186/s12884-019-2573-3)
Supplement: Supplementary file 2 — Additional file 2: Table S1. Questionnaire contents and data collection tools, Pelotas 2015. [file 12884_2019_2573_MOESM2_ESM.docx]

Supplementary Table 1s. Questionnaire contents and data collection tools, Pelotas 2015

| Modules | [Prenatal assessments*](file:///C:\Users\LENOVO\Documents\TESIS\Articulo%202\CORRECAO%201%20COAUTORES\Medical%20%20Care\BMC\Nuevo%20Hoja%20de%20cálculo%20de%20Microsoft%20Excel.xlsx#RANGE!tblfn1) | | | Postnatal assessments |
| --- | --- | --- | --- | --- |
|  |  |  |  |  |
|  |  |  |  |  |
|  | Initial | Main | Combined | Perinatal |
| Eligibility Criteria | X | X | X | X |
| Identification/Contact information | X | X | X | X |
| Socio-demographic characteristics | X | X | X | X |
| Maternal characteristics | X |  | X | X |
| Antenatal care | X | X | X | X |
| Reproductive health history/contraception |  |  |  | X |
| Pre-pregnancy health | X |  | X | X |
| Medicine use | X | X | X | [Xm](file:///C:\Users\LENOVO\Documents\TESIS\Articulo%202\CORRECAO%201%20COAUTORES\Medical%20%20Care\BMC\Nuevo%20Hoja%20de%20cálculo%20de%20Microsoft%20Excel.xlsx#RANGE!tblfn2) |
| Paternal characteristics | X |  | X | X |
| Physical activity questionnaire | X | X | X | X |
| Wellbeing |  | X | X |  |
| Alcohol use |  | X | X | X |
| Tobacco use | X | X | X | X |
| Illicit drug use |  | X | X | X |
| Employment assessment |  |  |  | Xm |
| Oral health |  | X | X | X |
| Pregnancy health | X | X | X | X |
| Edinburgh Postnatal Depression Scale |  | X | X | X |
| [Antenatal card image obtained#](file:///C:\Users\LENOVO\Documents\TESIS\Articulo%202\CORRECAO%201%20COAUTORES\Medical%20%20Care\BMC\Nuevo%20Hoja%20de%20cálculo%20de%20Microsoft%20Excel.xlsx#RANGE!tblfn3) | X | X | X | X |
| Anthropometry |  |  |  | [Xc](file:///C:\Users\LENOVO\Documents\TESIS\Articulo%202\CORRECAO%201%20COAUTORES\Medical%20%20Care\BMC\Nuevo%20Hoja%20de%20cálculo%20de%20Microsoft%20Excel.xlsx#RANGE!tblfn2) |
| Breastfeeding/Complementary feeding |  |  |  | X |
| Child Health/immunization |  |  |  | X |

^*^Antenatal care assessment instruments varied depending on the GA at enrollment. Women identified and enrolled before 16 weeks pregnancy answered the ‘initial assessment’ questionnaire and between weeks 17 and 24 a ‘main assessment’ questionnaire was applied (ideally at week 20 of pregnancy). Women enrolled after 16 weeks responded to the ‘combined assessment’ that consisted of a combination of the information collected in the ‘initial assessment’ and ‘main assessment’.

^m^Maternal; ^c^Child.

^#^Antenatal card contains information on laboratory results, clinical assessments, vaccines, medicines taken during pregnancy, family health history, last menstrual period (LMP), estimated delivery date based on LMP and based on Ultrasound.
