# Supplementary material for: Agreement of antenatal care indicators from self-reported questionnaire and the antenatal care card of women in the 2015 Pelotas birth cohort, Rio Grande do Sul, Brazil
Source: BMC Pregnancy Childbirth. 2019 Nov 8;19:410. doi: 10.1186/s12884-019-2573-3 (PMC6839160; doi:10.1186/s12884-019-2573-3)
Supplement: Supplementary file 3 — Additional file 3: Table S1. Questionnaire items and the antenatal care card variables. [file 12884_2019_2573_MOESM3_ESM.docx]

| **Self-report questionnaire**  **Service utilization**  How many antenatal care visits did you have?  Did the doctor or nurse ask you about the date of your last period? (yes/no)  **Clinical exams**  Did the doctor or nurse measure your weight? (yes/no)  Did the doctor or nurse measure your abdomen? (yes/no)  Did the doctor or nurse measure your blood pressure? (yes/no)  Did the doctor or nurse do the gynecological exam? (yes/no)  Did the doctor or nurse take cervical cancer prevention exam? (yes/no)  During your pregnancy, did you consult a dentist? (yes/no)  Did the doctor examine your breasts?  Did you have a positive syphilis test? (yes/no)  **Diseases during pregnancy**  Did you have high blood pressure? (yes/no)  Did you have anemia? (yes/no)  Did you have diabetes? (yes/no)  Did you have a urinary tract infection? (yes/no)  Did you have any sexually transmitted diseases like herpes, gonorrhea, trichomoniasis, genital warts, chlamydia, condyloma, or other? (yes/no)  Did you have a positive test for syphilis? * (yes/no)  **Vaccination- Supplements**  During your antenatal care, did you get the vaccine for tetanus toxoid or tetanus-diphtheria-acellular pertussis (Tdap)? (yes, or already vaccinated/no)**  During your antenatal care, did you get the vaccine for hepatitis B? (yes, or already vaccinated/no)**  Did the doctor give a medical prescription for anemia? (yes/no)  **Counselling**  Did the doctor or nurse counsel you about the risks of alcohol use during pregnancy? (yes/no)  Did the doctor or nurse counsel about the risks of smoking use during pregnancy? (yes/no)  Did the doctor or nurse counsel about physical activities such as walking? (yes/no)  * This question was coupled with the question about sexually transmitted diseases | **Antenatal care card *****  **Service utilization**  Number of antenatal care visits report  Report date of last menstrual period  **Clinical exams**  Weight measurement  Symphysis-fundal height measurement  Blood pressure measurement  Gynecological exam  Cervical cancer screening test  Dental exam  Breasts exam  Syphilis test  **Diseases during pregnancy**  Record of hypertension or prescription antihypertensive drugs  Record of anemia or level of hemoglobin < 11g/dL  Record of diabetes  Record of urinary tract infection, or positive urine culture results or positive for qualitative urine test.  Record of herpes, gonorrhea, trichomoniasis, genital warts, chlamydia, condyloma, and HIV  **Vaccination- supplements**  Record of vaccine for tetanus toxoid or tetanus-diphtheria-acellular pertussis (Tdap) **  Record of vaccine for hepatitis B**  Iron supplements prescription  **Counselling**  Risks of alcohol use during pregnancy  Risks of smoking during pregnancy  Physical activities such as walking  ** Any doses, booster, or already vaccinated were considered as: "yes"  ***The variables were recategorized as “yes” if they had at least one record on the card and “no” when there was no record |
| --- | --- |

Supplementary Table 1s. Questionnaire items and the antenatal care card variables.
